# Addressing the issue of bias in observational studies: Using instrumental variables and a quasi-randomization trial in an ESME research project

**DOI:** 10.1371/journal.pone.0255017

**Published:** 2021-09-15

**Authors:** Monia Ezzalfani, Raphaël Porcher, Alexia Savignoni, Suzette Delaloge, Thomas Filleron, Mathieu Robain, David Pérol, ESME Group

**Affiliations:** 1 Department of Biostatistics, Pôle de Biometrie, Institut Curie, Paris, France; 2 Université de Paris, Centre of Research Epidemiology and Statistics, INSERM U1153, Paris, France; 3 Department of Cancer Medicine, Institut Gustave Roussy, Villejuif, France; 4 Biostatistics unit, IUCT Oncopole, Toulouse, France; 5 Department of Research and Development, R&D Unicancer, Paris, France; 6 Department of Biostatistics, DRCI, Centre Leon Berard, Lyon, France; Goethe University Hospital Frankfurt, GERMANY

## Abstract

**Purpose:**

Observational studies using routinely collected data are faced with a number of potential shortcomings that can bias their results. Many methods rely on controlling for measured and unmeasured confounders. In this work, we investigate the use of instrumental variables (IV) and quasi-trial analysis to control for unmeasured confounders in the context of a study based on the retrospective Epidemiological Strategy and Medical Economics (ESME) database, which compared overall survival (OS) with paclitaxel plus bevacizumab or paclitaxel alone as first-line treatment in patients with HER2-negative metastatic breast cancer (MBC).

**Patients and methods:**

Causal interpretations and estimates can be made from observation data using IV and quasi-trial analysis. Quasi-trial analysis has the same conceptual basis as IV, however, instead of using IV in the analysis, a “superficial” or “pseudo” randomized trial is used in a Cox model. For instance, in a multicenter trial, instead of using the treatment variable, quasi-trial analysis can consider the treatment preference in each center, which can be informative, and then comparisons of results between centers or clinicians can be informative.

**Results:**

In the original analysis, the OS adjusted for major factors was significantly longer with paclitaxel and bevacizumab than with paclitaxel alone. Using the center-treatment preference as an instrument yielded to concordant results. For the quasi-trial analysis, a Cox model was used, adjusted on all factors initially used. The results consolidate those obtained with a conventional multivariate Cox model.

**Conclusion:**

Unmeasured confounding is a major concern in observational studies, and IV or quasi-trial analysis can be helpful to complement analysis of studies of this nature.

## Introduction

The goal of comparative clinical research studies is to compare treatment efficacy on a specified outcome; when an effect is found, it is crucial to be able to conclude on the causal effect of the treatment. Randomized controlled trials (RCTs) are the gold standard for identifying the causal impact of a treatment because randomization ensures that the patients’ baseline characteristics are well balanced between groups, by considering measurable or non-measurable variables. However, RCTs are not always feasible due to logistical or ethical constraints. Observational studies using routinely collected data (RCD) can provide an alternative source of data and play an important role in comparing the efficacy of pharmaceutical products and other healthcare interventions. These studies can complement RCTs by providing additional data from the real-life setting. However, a major challenge is the validity of RCD due to potential bias. Indeed, potential issues with RCD include lack of assignment, i.e. selection bias, causing imbalance between treatment groups with regard to prognostic factors, and lack of control, e.g. measurement errors, omitted variables or lack of measurement of all confounders. Confounders are variables that influence both the treatment assignment and the outcome. Some of the methods used to control the impact of confounders on the assessment of treatment effect include propensity scores, regression and matching [1–3]. These methods only control for measured confounders and do not control unmeasured confounders. Methods such as instrumental variables (IVs) [4–6] and quasi-trials [7] can be used to address the issue of unmeasured confounders. An instrumental variable has three key characteristics: i) it is highly correlated with treatment, ii) it only affects the outcome through treatment, and iii) it is not associated with unmeasured confounders after controlling for measured confounders. Provided these assumptions hold, cause-and-effect interpretations and estimates can be drawn based on analysis of RCD using IVs [7]. Different techniques are possible to analyze the IVs [2, 7, 8]: in this paper, we will present the two-stage residual inclusion (2SRI) approach. Concerning the quasi-trial method, the basic idea is to replace the treatment variable by a variable that creates a “pseudo” randomized trial (8). The Quasi-random methods (quasi = almost, but not completely random) used to allocate subjects to different arms of the trial (to receive the study medicine, or placebo, for example) using a method of allocation that is not truly random. This method has been rarely used in oncology context. The idea behind the quasi-randomized or pseudo-randomized trial is that the treatment or % treatment prescribed may vary from center to center and the results may also differ. For instance, in a multicenter trial, the treatment preference in each center can be informative, and then comparison of the results between centers or clinicians can be informative. A shared frailty model with random effects is used to account for the fact that patients were clustered within centers and that the treatment could vary between centers [9]. The shared frailty model is a random effects model where the frailties, i.e. latent multiplicative effects on the hazard function, are shared among the individuals of a center and are randomly distributed across the centers. The techniques used for IV or quasi-trial analyses are described in the Methods section.

This work was conducted on a retrospective database established in 2014 by the R&D Department of Unicancer, the national academic network of French Comprehensive Cancer Centers (FCCCs). They created the Epidemiological Strategy and Medical Economics (ESME) Research program to centralize real-world patient data in oncology. The program’s first project concerns the construction of a comprehensive database on patients with metastatic breast cancer (MBC), called the ESME MBC Database, using data obtained from the 18 FCCCs [10]. Among the outcomes studied, the ESME MBC Database (NCT03275311) has looked at Overall Survival (OS) in patients with HER2-negative MBC treated with first-line paclitaxel-based chemotherapy, with or without bevacizumab [11], referred to as the “Beva+Pacli/Pacli analysis” in this paper. OS adjusted for major prognostic factors, using multivariate Cox model, was significantly longer in the paclitaxel with bevacizumab group compared to the paclitaxel group [hazard ratio (HR) 0.672, 95% confidence interval (CI) 0.601–0.752; median survival time 27.7 versus 19.8 months]. The results were consistent in both supportive analyses, using a propensity score for adjustment or using a matching factor for nested case-control analysis. OS in patients receiving paclitaxel + bevacizumab was exactly as expected from clinical trials. However, the treatment effect estimate was discrepant with the results obtained in three earlier randomized comparative phase III trials, which led the FDA to withdraw approval for bevacizumab in this indication [12, 13]. Many factors could have contributed to this discrepancy, especially the fact that a majority of patients treated with paclitaxel alone benefited from the experimental treatment after progression (cross-over), leading to under-estimation of OS in the RCT, which was not the case in the real-life setting. Another factor could be the difficulty of extrapolating results from real-world cohorts due to the lack of randomization, as patients who are treated in real-life situations have highly differing and heterogeneous characteristics not found in the eligibility criteria in RCTs, and to the inability of the statistical methods used to control the eventual presence of bias related to unmeasured or imperfectly measured confounders [14].

Although unmeasured confounders are a major concern in observational studies in oncology, IV or quasi-trial analyses are rarely used. Our aim here is to present both the IV and quasi-trial methods, and to illustrate their use in the ESME MBC Database.

## Materials and methods

### Materials

The Beva+Pacli/Pacli study was a retrospective, registry-based study including 3426 patients, 2127 receiving paclitaxel and bevacizumab, and 1299 receiving paclitaxel as first-line chemotherapy. Details on the study have been previously reported [11]. The original analysis of OS was primarily based on a multivariable Cox model adjusted for major prognostic factors. The factors used for the adjustment were: time between metastatic diagnosis and index date, period of care, Scarff-Bloom and Richardson (SBR) grade III, age, triple-negative breast cancer status, type of metastases (visceral versus non visceral), number of sites, time between initial diagnosis and diagnosis of metastasis, and initial management (adjuvant chemotherapy and/or adjuvant endocrine therapy). It showed that OS was significantly longer with paclitaxel and bevacizumab compared to paclitaxel (hazard ratio [HR] 0.672, 95% confidence interval [CI] 0.601–0.752; median survival time 27.7 versus 19.8 months). Moreover, consistent results were obtained in the supportive analyses using a propensity score as adjustment variable and propensity score matching [3]. Note that neither of these approaches exactly estimated the same treatment effect. Propensity score matching aims at estimating a marginal hazard ratio (average treatment effect) in a given population, whereas the other methods estimate a conditional hazard ratio in the entire population [15, 16].

The patient records were fully anonymized before access to information. The Unicancer Institute review board approves the study.

### Statistical methods

In this work, we consider that the variable of interest is treatment; the response variable or the outcome is OS.

Two approaches will be presented: i) instrumental variables (IVs) [5, 6, 17, 18], and ii) quasi-trial analysis [7].

An application will be presented at the end of each approach using the Beva+Pacli/Pacli study.

### Instrumental variables analysis

**Fig 1:**
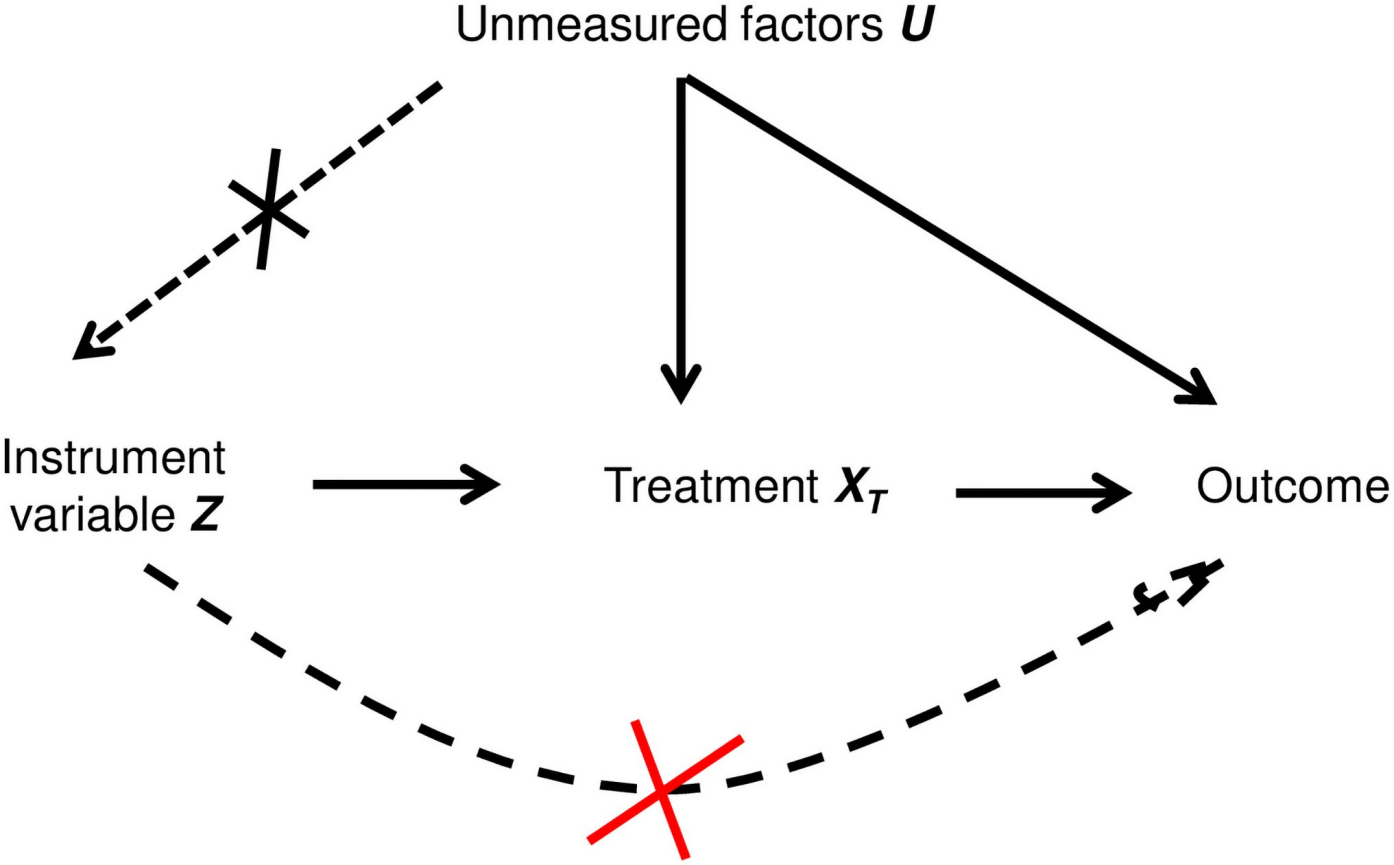
Assumptions of Instrument variable.

The basic idea of IV approach is to use an additional variable, called an “instrumental variable” or simply an “instrument”. As shown in Fig 1, a valid IV must satisfy three assumptions. Firstly, it has a causal effect on treatment, i.e. the IV causes a change in treatment assignment. This assumption can be confirmed by measuring the association between the IV and treatment. Secondly, it does not independently affect the response variable, i.e. the IV does not directly impact the response variable, but only through its impact on the treatment. This is called exclusion restriction (ER). For instance, in many clinical problems, there are other treatments that could be used alongside the treatment under study. If a proposed IV is associated with these concomitant treatments and they causally affect the outcome, then the ER would be violated. Thus, exploring the association between the proposed IV and concomitant treatments can help determine whether the ER is violated [17]. Thirdly, the IV is not associated with measured or unmeasured patient health status (Fig 1). For instance, if a measured confounder is only a proxy for a true confounder, then an association between the IV and this measured confounder suggests that there will be an association between the IV and the unmeasured part of the true confounder [17]. To assess the third assumption for the IV (Fig 1), we checked the imbalance of measured covariates across levels of the IV as well as their association. The association between IVs and measured covariates was obtained using a chi-square test or analysis of variance (ANOVA).

To summarize, the inherent idea of IV is to remove any variation in the treatment that is not related to unmeasured covariates and to use it to estimate the casual effect of the treatment.

Two main approaches have been proposed to conduct IV analyses: two-stage predictor substitution (2SPS) and two-stage residual inclusion (2SRI) [5, 6]. Under the 2SPS approach, the first stage model yields the predicted value of treatment as a function of an instrument and covariates, and in the second stage model for the outcome, this predicted value replaces the observed value of treatment as a covariate. Under the 2SRI approach, the first stage is the same, however the residual term of the first stage regression is included in the second stage regression, retaining the observed treatment as a covariate. Both 2SPS and 2SRI yield consistent estimates for linear models [5], however, recent work has shown that the approaches lead to inconsistent estimates for nonlinear models, in particular the Cox model [17–19]. Martinez-Camblor et al. showed that instrumenting an endogenous treatment induces an unmeasured covariate, referred to as an individual frailty in survival analysis, which if not accounted for leads to bias [4]. They proposed a new and more adequate procedure for the Cox model using 2SRI with an individual frailty, denoted as 2SRI-frailty. In this approach, the Cox model is introduced with an individual frailty term in the second-stage estimation, as shown in the equations below.

Let p be the probability to receive the Beva+Pacli treatment, and the variables X, X_T_, and Z refer to the measured confounders, the treatment, and the IV, respectively.


$$logit\left(p\right)=\alpha +\beta_{X}X+\;\gamma \;Z$$

We then compute the residuals $\hat{\text{R}}$ These residuals contain all the information about the unmeasured variables related to the treatment assignment and unrelated, and also contain white noise corresponding to a random factor affecting an individual’s treatment selection, which can be handled by specifying an individual frailty in the Cox model:

$$\lambda \left(t\right)=\phi \lambda_{0}\left(t\right)\text{exp}\left(\alpha +\beta_{IV}X_{T}+\;\beta_{X}X+\;\beta_{R}\;\hat{\text{R}}\right),$$

where *ϕ* is the individual frailty term, and *β*_*IV*_ is the treatment effect estimated using the IV approach. We assumed that the frailty term *ϕ* was disturbed according to a Gamma distribution.

Following these recommendations, the 2SRI-frailty is applied to the Beva+Pacli/Pacli analysis. The Cox model used in the original paper by Delaloge *et al*. was adjusted on pre-chemo period, period of care and SBR grade III, and was stratified on age, triple negative status, metastasis type, number of metastasis site, time of metastatic disease, adjuvant chemotherapy and hormone therapy. The same covariates were used for the 2SRI-frailty.

### Application

To analyze the Beva+Pacli/Pacli data, we used two different candidate IVs. First we used the activity level of the FCCC (denoted as IV1), in three strata as presented in the ESME dataset, i.e. reference strata < to 500 patients per center, between 500 and 800 patients per center, and > 800 patients per center. These three strata were available in the database, with the patient repartition, in each stratum, equal to 16%, 32%, and 52%, respectively. Secondly, we used the treatment preference in the center (denoted as IV2), treated as a continuous variable, as presented in the ESME dataset.

Intuitively, both IVs are expected to affect the treatment assignment, and should not be influenced by the data on a particular patient. The choice of these variables is based on the natural variation in medical practice at each IV level [17]. As previously stated, it is important to check that these variables have a direct effect on the treatment using statistical tests. For the second feature of the IV assumptions (ER is violated), we checked the association between the proposed IV and concomitant treatments. To assess the third feature of the IV assumptions (see Fig 1), we checked the imbalance of measured covariates across levels of the IV as well as their association. The association between the IVs and measured covariates was obtained using a chi-square test or ANOVA.

### Quasi-trial analysis

As with IV estimation, the idea of quasi-trial analysis is to create a “superficial” or “pseudo” randomized trial using variables that control for the bias inherent in nonrandomized comparisons [7]. For instance, the treatment preference in center can be considered. Indeed, comparisons of results between centers can be informative since it is likely that differing preferences exist in practice. Although it shares some conceptual similarities with IV analysis, analysis of this variable in the Cox model differs from that in the IV approach. For the quasi-trial analysis in the Cox model, we replaced the treatment variable by considering the center-specific percentage of patients treated with Beva+Pacli. This model compares the HR of a hypothetical patient treated at a center in which all patients were treated with Beva+Pacli with an identical patient treated at a center in which no patients were treated with Beva+Pacli. However, in practice, it is impossible to expect 100% Beva+Pacli in all centers. A shared frailty model with center-specific random effects was used to account for the fact that patients were clustered within centers and the percentage of the treatment Beva+Pacli could vary between centers, as

$$\lambda_{ij}\left(t\right)=\phi_{j}\lambda_{0}\left(t\right)\text{exp}\left(\alpha +\beta_{T}X_{T}+\;\beta_{X}X_{ij}\right),$$

where *ϕ*_*j*_ represents the frailty term shared for all patients *i* in each center *j*, *and β*_*T*_ is the effect treatment. We assumed that the frailty term *ϕ* is distributed according to a gamma distribution. In this case, the shared frailty model is then a random effects model where the frailties (latent multiplicative effects on the hazard function) are shared among the individuals of a center and are randomly distributed across the centers. The adjusted HR was calculated for class >50%, which included centers with a preference for the treatment Beva+Pacli above 50%. The class (≤50%) is the reference group used in a Cox regression model, adjusted on all significant baseline factors used in the original analysis [11].

A measurement error linear regression model was fitted between the treatment classes and the adjusted HR, with allowance for the estimation errors in both. The simulation extrapolation (SIMEX) method was used to ensure convergence of the model. The SIMEX method reduces the bias induced by measurement error by establishing a relationship between measurement error–induced bias and the variance of the error. The SIMEX model is considered to be a relevant tool for correcting effect estimates in the presence of additive measurement error on the explicative variable (here, the percentage of patients with treatment in each center). As this model is not applied with only two classes, we have proposed to measure errors using three classes of reintroduction of Beva+Pacli: i) class 1: ≦50%, ii) class 2: [50–66], and class 3: ≧66%. OS curves were estimated by reintroduction class (using the two classes of interest: ≤50% versus ≥50%) both as Kaplan-Meier estimated curves and as Cox model predicted curves.

### Application

To analyze the Beva+Pacli/Pacli data, the treatment preference by center has been used. In fact, it is recommended to use a variable that depends partly on physician preference, which suggests the possibility that individual physicians could be used as the basis of a natural experiment. This variable reflects the preference for certain treatment options in different centers. It was grouped into two classes of percentages of patients treated with Beva+Pacli (≤50% versus ≥50%).

## Results

### Instrumental variables analysis

To assess the first feature of the assumptions for IV (Fig 1), we proposed to use a univariate logistic model where the treatment was explained by the candidate IV1. There was a significant association between this IV1 and the treatment. The global Wald test was significant (p-value = 0.0014). Using a multivariate logistic model adjusted on prognostic covariates as used in the Beva+Pacli/Pacli analysis, the Wald test corresponding to the IV1 remained significant with a global p-value of 0.0014. In addition, a Cramer V test was used to assess the intensity between the IV1 and the treatment, and the p-value was 0.05, showing a weak association.

The imbalance of measured covariates was studied across each level of IV1. The distribution of variables was not balanced across the different strata of the IV for all covariates. The covariates for which the imbalance was not respected are shown in Table 1. This suggests that there may be residual confounding due to these covariates. The association between the IV and measured prognostic covariates was not statistically significant (p-value>0.05) for period of care, age class, triple-negative breast cancer status, number of sites class, and type of metastases (visceral versus non visceral). Using the 2SRI-frailty method with IV1, the OS adjusted for major prognostic measured and non-measured factors was longer in the paclitaxel and bevacizumab group compared with paclitaxel [hazard ratio (HR) 0.90, 95% confidence interval (CI) [0.22–3.73].

**Table 1:** Imbalance between measured covariates and IV1.

	Number of patients at each stratum	Period of care (classes)		Time between metastatic diagnosis and index date (months)		
Covariates IV1		2008–2010	2011–2013	<2	[2–6]	>6
<500	561	240	321	424	73	64
[500–800]	1093	436	657	708	190	195
>800	1772	689	1083	1265	301	206

For the treatment preference in center, the Wald test corresponding to the IV2 was significant with a global p-value<0.0001, in univariate and multivariate logistic models adjusted on prognostic covariates. Using the 2SRI-frailty method with IV2, the OS adjusted for major prognostic measured and non-measured factors was longer in the paclitaxel and bevacizumab group compared with paclitaxel [hazard ratio (HR) 0.65, 95% confidence interval (CI) [0.39–1.08]. The HR was similar to that obtained in the Beva+Pacli/pacli analysis, which was 0.67 with a 95% CI of 0.60–0.75.

The association between the IV and measured prognostic covariates was not statistically significant (with p-value>0.05) for time between metastatic diagnosis and index date, SBR grade III, triple-negative breast cancer status, type of metastases (visceral versus non visceral), number of sites, and initial management (adjuvant chemotherapy and/or adjuvant endocrine therapy).

We applied the 2SRI-frailty method by including both candidate IVs. Using multivariate logistic model adjusted on prognostic covariates and both IVs, the Wald test corresponding to IV1 was non-significant (p-value 0.88), and significant for the IV2 with a p-value<0.0001. Using the 2SRI-frailty method with both IV, OS adjusted for major prognostic measured and non-measured factors was significantly longer in the paclitaxel and bevacizumab group compared with paclitaxel [hazard ratio (HR) 0.56, 95% confidence interval (CI) [0.33–0.94]. The results were close to those obtained using only IV2 in the model.

### Quasi-trial analysis

The treatment preference, computed in each center, varied from 45.5% to 81.3%, with a mean of 62.1% and a median of 59.0%. The shared frailty model with center-specific random effects was fitted using the same baseline factors plus the treatment preference, computed in each center. The treatment preference had a significant association with OS (HR = 0.18, 95%CI = 0.04–0.75). This model indicated that a hypothetical patient treated at a center in which all patients were treated with Beva+Pacli would have a significantly reduced risk of death by approximately 82 per cent compared with an identical patient treated at a center in which no patients were treated with Beva+Pacli. As this case is not possible in practice, we proposed to consider the percentage of patients with Beva+Pacli in two classes ≤50% versus >50%.

Median survival time was 18.7 months in centers in which the percentage of treatment was ≤50%, and 26 months in centers in which the percentage of treatment was >50% (see Table 2).

**Table 2:** Median survival and HR for centers grouped by % of patients treated.

Patients on Beva+Pacli (%)	Patients on Beva+Pacli (mean %)	Centers (N)	Patients (N)	Median survival (months)	OS HR	95% CI	
≤50	47.1	4	435	18.7	1	Reference	
>50	64.3	15	2991	26.1	0.74	0.65	0.84

We compared naïve regression and the Simex model to quantify potential measurement errors related to adjusted HR and the percentage of patients treated. As mentioned previously, since this approach is not applicable for a variable with two classes (≤50% vs >50%), we proposed to measure the error using three classes of the percentage of patients treated, i.e. ≤50%; [50%-66%] and ≥66%. The HR was 0.71 and 0.75 in the classes [50%-66%] and ≥66%, respectively. The class ≤50% was the reference group. The two HR were similar. The Simex model applied to these data yielded results similar to those with the naïve model. This made no allowance for the fact that the percentage of patients treated with Beva+Pacli and the HR were both estimated with error.

The Cox model adjusted on all predicted factors (listed in the final model) and including the percentage of patients treated in two classes was considered. The HR was 0.74, with a 95% CI of 0.65–0.84.

## Conclusions and discussion

Evaluating treatment efficacy and identifying the causal relationship between exposure and disease course are the objectives of many clinical studies or field clinical research. Despite the fact that IV or quasi-trial analyses are rarely used in oncology, these methods provide a relatively simple way to analyze OS in studies where unmeasured confounders are present. This type of analysis requires the use of a valid IV, which can be difficult in practice for two main reasons. First, it may be difficult to find an IV in the database of the study concerned since all major confounding covariates are often collected and, secondly, in practice, it may be difficult to check the various assumptions for the validity of an IV [17–22].

Concerning the first point, the difficulty resides in finding an IV collected in the data. Use of various IVs has been recommended depending on the context [17, 18]. For instance, it was recommended to use “distance to specialty care provider” when comparing two treatments where one is provided only by specialized care providers and the other only by general providers. Preference-based IVs are recommended if variations in medical practice patterns at the geographic, hospital or physician level are available and collected in the data. Such variations can dictate how drugs or medical procedures are used. In our application, both IVs, the activity level of FCCC and the treatment preference by center, were preference-based as reported in the database, and were expected to affect the treatment assignment. As the treatment preference, the activity of the FCCC can affect the treatment assignment. In fact, we can expect that large centers will be more likely to follow innovations and prescribe new treatments. So, this variable reflects the preference for certain center options according to the different centers.

Concerning the IV assumptions, if assumptions ii) and iii) (Fig 1) do not hold, an IV analysis can yield a biased estimate of the treatment effect. One way to jointly test hypotheses (i) and (ii) is to find a sub-population in which the relationship between the IV and treatment is broken, and then check whether or not the proposed IV is associated with the outcome. If this is the case, it means the IV is associated with unmeasured confounders or directly with the outcome through variables other than treatment [17]. These assumptions could not however be readily or entirely be tested with our data. For instance, to check the second feature of the IV assumptions, concomitant treatment to chemotherapy were not reported in our application, so they could not be taken into account in the analysis. This is a limitation of our analysis, and was also a limitation of the original Beva+Pacli/Pacli study. However, it is recommended to examine the relationship between the IV and the measured patient characteristics [17, 18]. Another critical issue is the strength of the relationship between the IV and treatment. The more strongly an IV is related to treatment, the more efficient the estimator will be, i.e. the smaller the standard error. The magnitude of the bias also depends on the strength of the association between the IV and treatment, with weaker IVs yielding more biased estimates [17, 18].

In addition to checking the validity of the IV, unmeasured confounding can be a major concern in investigator choices. In our application, treatment was intentionally chosen by physicians and potentially by patients, however there is often substantial unmeasured confounding from unmeasured indications or severity. As discussed by Baiocchi et al., an IV analysis can therefore be helpful for this kind of study. When an IV is available, even if it is not perfectly valid, an IV analysis or a sequence of IV analyses with various IVs can provide very helpful information about treatment effect [17, 22]. For studies in which unmeasured confounding is not a major concern and no strong IV is available, the authors advise investigators to consider IV analyses as secondary or sensitivity analyses.

IV analysis of survival data is also not straightforward. “Classical” methods (2SPS/2SRI) have been challenged, and specific approaches have recently been proposed. The Martinez-Cambor approach is applied to estimate an HR, which is the same measure of treatment effect as in the original data analysis. However, this does not mean that other approaches to IV analysis of survival data could not be useful for analysis of the Beva+Pacli /Pacli study. Results with the IV1 were not concordant with those obtained in the Beva+Pacli /Pacli analysis, with a large confidence interval. The Cramer V test showed a weak association between treatment and IV1. Baiocchi et al. have demonstrated that when the IV is weak, even if it is a valid IV, treatment effect estimates based on IV methods have some limitations, such as large variance even with large samples, which can lead to bias in treatment effect estimates [17, 22]. We note that an IV is considered to be strong if it has a strong impact on treatment choice and a weak IV if it only has a slight impact. Results with the IV2 were consistent with those obtained in the Beva+Pacli /Pacli analysis, with an HR of 0.65 and a 95% CI of 0.39–1.08. The HR is close to the one obtained previously, i.e. 0.67, with a 95%CI of 0.60–0.75. Results are summarized in Table 3. Obtaining consistent results may be due to the fact as the major confounders in breast cancer are becoming well known and not as many unmeasured (major) confounders as that.

**Table 3:** OS HR, and 95% CI for the different approaches using Beva+PAcli database.

	OS HR	95% CI
Multivariate Cox model (original paper by Delaloge *et al*.*)*	0.67	0.60–0.75
Instrument variable (using IV1*)	0.90	0.22–3.73
Instrument variable (using IV2**)	0.65	0.39–1.08
Instrument variable (using both IV1 and IV2)	0.56	0.33–0.94
Quasi-trial approach (IV2)	0.74	0.65–0.84

*: IV1: the activity level of the FCCC.

**: IV2: treatment preference by center.

The quasi-trial analysis is a second way to treat unmeasured confounders in RCD. Its use requires finding a variable that depends partly on physician preference, which suggests the possibility that individual physicians could be used as the basis of a natural experiment. Treatment preference by center was considered. This variable reflects the preference for certain treatment options in different centers, and was grouped into two classes of percentages of patients treated with Beva+Pacli. We noted that the HR was associated with the treatment preference by center, and therefore can reflect the treatment effect because if the treatment preference in the center is higher than 50%, the probability of treating a patient with Beva+Pacli is high. This result is concordant with the result in the initial Beva+Pacli/Pacli analysis, showing a significant effect (see Table 3).

We note that the different analyses accounted for baseline confounding, not time-dependent confounding. Indeed, in the Beva+Pacli trial, the patients are allocated to the Pacli or Beva+Pacli arm for their first line treatment, and all characteristics are measured and taken at baseline (not varying across time). However, in some context taking onto account time-dependent confounding can be an issue. Recently, the residual inclusion methods have been adapted to additive hazard models for censored survival data by taking into account time-dependent covariate effects [23].

In fact, initially the two-stage least squares method, where the first stage consists of a linear model for the confounded exposure given the IV and other observed covariates, accounted for time-independent covariates in semi-parametric additive hazard model developed by Li et al. [24]. Then, a similar 2SLS method for continuous instruments was proposed by Tchetgen et al. [25] for the non-parametric additive hazard model of Aalen [26], where all covariate effects are allowed to be time dependent. Brueckner et al. have demonstrated that the 2SRI method avoids the bias of 2SLS when censoring depends on the exposure and when the first stage is a non-linear model through a simulation study [27]. In this analysis, we focused on the construction of the IC and how it can be considered using a proportional hazards model, the application of semi-parametric additive models with time-dependent variables for the Quasi-trial approach will be the topic for a further paper.

Finally, large RCTs are critical to addressing treatment efficacy, as well as clinical questions on major patient outcomes such as survival, however real-world data from robust RCD provide valuable data that complement RCTs. Taking into account that unmeasured confounding is a major concern in RCD, an IV or quasi-trial analysis can be helpful to complement this kind of study. While the IV method is widely used with observational data in econometrics, its application in oncology data is rare. The quasi-trial analysis has, to our knowledge, been proposed in only one case in oncology [7]. This paper shows how the two methods can be applied in practice in oncology, and presents their limitations. No comparison between the two methods was made, however, assessing different analytic approaches to removing the effects of bias in observational studies, such as multivariable model risk adjustment, propensity score risk adjustment and propensity-based matching will be the topic of further research using simulation studies.
